# Navigating a transforming landscape: the evolving role of pharmacovigilance physicians in drug development and implications for future challenges and training requirements

**DOI:** 10.3389/fdsfr.2023.1257732

**Published:** 2023-09-06

**Authors:** Tarek A. Hammad, Salman Afsar, Herve Le-Louet, Veronique F. Kugener

**Affiliations:** ^1^ Head of Medical Safety, Marketed Products, Global Patient Safety Evaluation, Takeda Pharmaceuticals Inc, Cambridge, MA, United States; ^2^ Medical Safety Assessment Physician and Program Lead, Worldwide Patient Safety, Bristol Myers Squibb, Princeton, NJ, United States; ^3^ Head of PV Policy Intelligence Strategy, Global Patient Safety Evaluation, Takeda Pharmaceuticals Inc, Cambridge, MA, United States; ^4^ President of the Council for International Organizations of Medical Sciences (CIOMS), Geneva, Switzerland; ^5^ Head of Global Patient Safety Evaluation, Takeda Pharmaceuticals Inc, Cambridge, MA, United States

**Keywords:** pharmacovigilance, role of pharmacovigilance physicians, safety physicians, evidence-based medicine, enhanced safety surveillance technologies, pharmacovigilance physician training requirements

## Abstract

Contrary to the famous quote from Voltaire, “The art of medicine consists in amusing the patient, while nature cures the disease”, medicine has evolved since the 17th century into a multi-faceted scientific field facilitating healing and improving overall wellbeing. One rapidly evolving area within this field is drug safety, also known as pharmacovigilance (PV). PV identifies and evaluates potential risks throughout the life cycle of the drug, minimizing patient exposure to harmful effects and guiding appropriate risk mitigation and management strategies. Timely identification and mitigation of risks not only contribute to patient safety but also allows maximum therapeutic benefits while curtailing economic burden associated with adverse events. In the evolving landscape of drug safety, the role of the PV physicians has emerged as an integral component of drug development. This paper aims to explore the evolving nature of PV physicians’ roles in drug development, highlighting changing landscape in drug development and safety monitoring and attendant changes and advancements in responsibilities, scope, and training implications. To be well-rounded, PV physicians are encouraged to strive to undergo relevant training and education. This would enable them to leverage pertinent complementary fields of science by developing the proficiency to ask the right questions, acknowledge multidisciplinary perspectives, and interpret the overall evidence. While on-the-job training is valuable for gaining experience, building a future safety workforce necessitates more targeted efforts, especially considering that medical school curricula may not readily emphasize the development of skills required for successful PV physician roles. Therefore, academic centers, pharmaceutical companies, and regulatory agencies should increase collaboration to establish hands-on training opportunities through post-doctoral, internship, and fellowship programs, in order to meet the growing demand for well-trained PV physicians.

## 1 Introduction

Pharmacovigilance (PV), an essential discipline in drug development and healthcare, encompasses the “detection, assessment, understanding, and prevention of adverse effects or any other medicine/vaccine related problem.”^1^ Over the years, PV has evolved to include not only prevention but also the minimization, mitigation, and impact assessment of adverse effects. Its primary goal is to ensure patient wellbeing and safeguard public health by identifying and evaluating potential risks associated with pharmaceutical products. Additionally, PV plays a significant role in drug access policies and health technology assessments. Robust PV measures help understand and characterize the safety profile of a drug, support informed decision-making by healthcare professionals and regulatory authorities, and foster public confidence in the healthcare system. Further, PV plays a critical role in the benefit risk (BR) assessments in the pre- and post-marketing phases and critically contributes to the ultimate benefit of patients.

It was not until the emergence of modern medicine and advancements in pharmacology that the need for dedicated professionals to oversee and manage drug safety became apparent (Fornasier et al., 2018). The thalidomide tragedy in the 1950s and 1960s played a pivotal role in shaping drug safety practices. The widespread use of thalidomide as an anti-nausea medication during pregnancy led to severe birth defects (Kim and Scialli, 2011), highlighting the necessity for robust safety monitoring and regulation. Consequently, regulatory agencies worldwide continue to strengthen oversight and introduce rigorous safety assessment processes.^2^ ^,^ ^3^ ^,^ ^4^

PV physicians, also known as safety physicians, specialize in detecting, assessing, and managing device, vaccine, and drug-related risks. They identify and report adverse events, conduct safety signal detection, and analyze data to assess risks and ensure appropriate risk management strategies and communications. As PV practices evolved, PV physicians emerged as vital contributors to drug development efforts throughout a product’s lifecycle. The increasing recognition of PV physicians as integral members of the development teams reflects the growing importance placed on proactive safety signals detection, monitoring, and risk management. This paper aims to explore the evolving nature of PV physicians’ roles in drug development, highlighting changing landscape in drug development and safety monitoring and attendant changes and advancements in responsibilities, scope, and training implications.

## 2 Changing landscape in drug development and safety monitoring

The landscape of drug development is currently undergoing a significant transformation driven by insights into molecular disease mechanisms and advances in drugs, biologics, and genetic therapies. This shift has led to creation of innovative therapies based on our comprehension of disease pathophysiology at molecular level. Central to this transformation is the recognition of the importance of anticipating and planning for early collection of safety-related data in drug development. To achieve this, a deeper understanding of related scientific disciplines is essential because integrating diverse data types and sources of evidence has become crucial in drug safety. These changes are shaping the evolving needs for the expertise required of PV physicians. Ongoing education, training, and professional development are essential for staying current with scientific advancements, regulatory changes, and technological progress (Kugener et al., 2021). This would ensure accurate and timely safety profile assessment throughout the product lifecycle. Here are some key drivers steering this evolution.

1. Advancement of Medical Sciences with Increasing Complexity and Volume of Drugs and Related Data: Modern drug development involves complex molecules, including biologics, gene therapies, cell therapies, and nanomedicines (Ioannidis et al., 2018; Mendicino et al., 2019). These advanced therapies pose unique safety challenges due to their novel mechanisms of action. For example, gene therapy products are designed to provide benefit through a long-acting or permanent mechanism of action, and the resulting long-term exposure may place patients of investigational studies at increased risk for delayed adverse effects requiring additional PV activities (Kugener et al., 2021). Further, the advent of these new therapies adds significance to the role of translational science, pertaining to findings from *in vitro* and *in vivo* preclinical studies, in understanding and predicting safety findings. There is also a need to better understand the scientific underpinnings of important safety findings encountered with new targeted and immune therapies, which are increasingly being studied. Mechanistic uniqueness of some of these molecules and the nature of associated risks might entail the need for better understanding of the risk interval and follow up duration requirements. For example, setting up risk interval (i.e., the interval within which the toxicity is expected) and follow up duration (i.e., the time needed for the toxicity to materialize) for drugs that may trigger immune mediated reactions after a predictable period of time driven by their mechanism of action^5^ can significantly facilitate timely identification of relevant cases. Many of these scientific areas require in-depth training and understanding. Having this knowledge would reduce the resources needed during drug development investigating safety signals without compromising the BR profile.

Moreover, with expanding pipelines and a better insight of diseases, there is a significant surge in the volume and complexity of efficacy and safety data. This necessitates the adoption of advanced technology platforms to create robust data management systems and innovative analytical tools for data analysis and interpretation. PV physicians should be trained to utilize and adapt to the evolving technology for effective monitoring and evaluation of the safety profiles of these complex products.

2. Precision Medicine and Pharmacogenomics^6^: The increasing focus on precision medicine and pharmacogenomics, which considers an individual’s genetic makeup for treatment decisions, impacts drug safety practices. Understanding how genetic variations influence drug responses and adverse events might be essential for optimizing drug safety and minimizing risks. For example, response-guided therapy (Kwo, 2011), where treatment decisions are based on how rapidly Hepatitis C virus (HCV) responds to treatment, has already been reported to be successful for genotype 1 HCV infection (Etzion et al., 2020). This approach avoids exposing patients to unnecessary adverse effects of therapy. Hence well-trained safety physician, who is knowledgeable about basic pharmacogenomic principles and their implications for drug safety monitoring, is integral for understanding and managing the safety profile.
3. Enhanced Safety Surveillance Technologies: Advancements in technology have led to the development of advanced safety surveillance tools and techniques. Data mining algorithms, natural language processing (NLP), machine learning, and artificial intelligence (AI) are being proposed to analyze large volumes of data, such as electronic health records, social media, and spontaneous reporting databases, to identify potential safety signals (Danysz et al., 2019; Murali et al., 2019; Liang et al., 2022).^7^ ^,^ ^8^ Even regulators are contemplating the potential role of these technologies (Ball and Dal Pan, 2022).^9^ PV physicians might be able to leverage these technologies to streamline processes, improve efficiency, and enhance the overall effectiveness of PV activities in analyzing data, identifying patterns, assessing causality and prediction of safety risks.

Automation in PV might, at least in theory, encompass various areas, such as case processing, signal detection, data extraction, reporting, quality control, and literature screening. For example, NLP techniques are used to automate the extraction and processing of relevant information from unstructured text in adverse event reports and patients’ medical records (Sheikhalishahi et al., 2019). Although this technology is supposed to improve the speed and accuracy of case intake, coding, and data entry processes, reducing manual effort, and enabling faster analysis, it might be challenged to some extent by the complexity of the medical field and its terminology. This might explain why it is still not widely utilized yet.

Moreover, with the widespread use of social media platforms, some PV teams are exploring the use of AI and NLP techniques to monitor social media data for potential safety signals (Bacilic et al., 2020). However, crucial pieces of information that are needed for the assessment might not always be available, e.g., germane aspects of patient medical history. Other researchers are purporting that advanced analytics and machine learning models can help predict the likelihood and severity of adverse events associated with specific drugs or patient populations, using non-clinical lab and animal data with what is known about the mechanism of action of the products (Ietswaart et al., 2020).

AI methodology is known for its ability in pattern recognition that has been shown to be applicable in the medical field (Afifi et al., 1995; Hammad et al., 1996; Hammad, 1998). In practice, identifying patterns and trends through these tools might help devise monitoring and mitigation plans by gaining more insight about the attributes of encountered Adverse Drug Reactions (ADRs). For-example, C_max_ is a pharmacokinetic measure used to determine drug dosing as it reflects the highest concentration of a drug in target organ after a dose is given.^10^ If certain ADR is identified with specific time-to-onset in correlation with C_max_, then appropriate monitoring and dosing can help mitigate the risk effectively. These can be useful tools for the PV physician specially when studying complex drugs such as antibody drug conjugates. Therefore, they must stay updated on these technological advancements and acquire the necessary skills to leverage these tools effectively and know its attendant limitations.

4. Increased Regulatory Scrutiny and Complexity in Global Regulations: Regulatory agencies worldwide are implementing changes to enhance drug safety monitoring and post-marketing surveillance that might require sophisticated study design and methodologies. These changes include stricter pre- and post-marketing reporting and surveillance requirements, risk management plans as well as mandates of assessment of effectiveness, signal detection methodologies, post authorization safety studies, and post-marketing requirements.^11^ ^,^ ^12^ ^,^ ^13^ These evolving trends introduce additional intricacies in complying with global regulations, necessitating comprehensive knowledge of different regulatory frameworks and the ability to adapt quickly to the ever changing and expanding landscape. Thorough and updated knowledge and understanding of evolving regulations is crucial for PV physicians to ensure compliance and adapt their practices accordingly. Therefore, there is a growing need for, PV physicians to actively participate in regulatory discussions, contributing their expertise to shape and harmonize policies and guidelines through the public commenting process offered by many regulatory agencies.
5. Emphasis on Patient-Centric Approach in Drug Safety: Patient engagement in PV is increasingly recognized as important aspect (Smith et al., 2016; Younus et al., 2023).^14^ Patients are ever more empowered to report adverse events (AEs) directly to regulatory authorities or through patient support programs. Patient reported events require critical thinking skills for accurate interpretation of the reported information. This additional source of safety data also requires PV physicians to promptly follow up with AE reporters with appropriately phrased questions, preferably using targeted questionnaires, to facilitate data gathering and appropriate analysis of the reported AEs.

Moreover, the patient-centric approach is being advocated in the context of BR assessment through the meticulous collection of patient preferences (Smith et al., 2016; Janssens et al., 2023). PV physicians are encouraged to actively collaborate with healthcare professionals, patients, and advocacy groups to ensure patient-centric approaches to drug safety. Healthcare professionals provide real-world insights into the safety and effectiveness of drugs in clinical practice. Patient involvement is increasingly recognized as essential, as patients provide unique perspectives and contribute to adverse event reporting. Advocacy groups represent patient interests and raise awareness of safety concerns. Collaboration with these stakeholders helps PV physicians gain diverse perspectives and understand drug safety comprehensively, supporting patient-centric approaches to PV. Nonetheless, In some instances, inaccurate data might create fears and contribute to the spread of misinformation, which can be counterproductive during health crises, such as creating unfounded fears against vaccines. Additionally, the large volume of unrelated cases of adverse events in extensive safety databases can lead to challenges in the efficiency of the signal detection process. Overall, it is crucial to strike a balance between the valuable insights gained from collaboration with these stakeholders and ensuring the accuracy and evidence-based nature of safety information. This approach helps to foster trust, provide reliable information, and address public concerns effectively in a timely fashion.

6. Leveraging Real-World Evidence: Real-world evidence (RWE) refers to information drawn from the analysis of routinely collected real-world data (RWD) pertaining to a patient’s health status or healthcare delivery. This data comes from sources beyond traditional clinical trials, encompassing registries and other resources (Cave et al., 2019). There is a growing emphasis on leveraging RWE alongside traditional clinical trial data in PV (Hammad et al., 2008; Hammad et al., 2013; Margulis et al., 2013; Pinheiro et al., 2013) and benefit risk assessment (Pinto et al., 2019; Radawski et al., 2020). The idea is to harness big data for post-marketing surveillance of rare adverse events as well as to evaluate long-term safety profiles of drugs. Studies exploring RWE’s application in regulatory decision-making demonstrate its increasing use in supporting medicinal product applications (Pontes et al., 2018). RWE would play a significant role in fulfilling the increasing requests for Post-Authorization Safety Studies (PASS),^15^ which aim at evaluating the safety of drugs in real-world settings following their approval. Post-marketing requirements and commitments (PMRs/PMCs)^16^ are another important aspect of pharmacovigilance. These activities are often conducted as a regulatory requirement, sometimes as a part of risk management plans. Their aim is to gather additional safety data, assess the long-term effects of a product, detect rare adverse events, and/or evaluate the effectiveness of risk minimization strategies. The value of this source of data is even more pronounced in case of rare diseases where the number of patients studied during development is small. In this case, patient registries, for instance, might offer solutions by providing data on treatment patterns and clinical outcomes (Jonker et al., 2022). The findings from all these activities can contribute to regulatory decision-making, label updates, and the implementation of additional risk management measures to ensure patient safety.

RWE sources, such as electronic health records, administrative claims databases, and patient registries might provide valuable insights into drug safety in diverse patient populations. To provide guidance to the field, the Council for International Organizations of Medical Sciences (CIOMS) has developed a draft consensus report on the use of Real World Evidence (RWE) for decisions about drug authorization, reimbursement, and clinical use.^17^ One approach is the traditional use of RWE through epidemiological studies, but another development pertains to the potential utility of the advanced safety analytics tools (e.g., AI) directly to integrate the large volumes of medical data. Integrating and analyzing these data require updated knowledge and analytical capabilities. Although the PV physicians might not conduct the actual analysis, they still need to understand the scientific underpinnings to be able to ask the right question and to interpret the research findings. These skills allow PV physicians to appreciate acceptable evidentiary threshold to account for the limitations and uncertainty in evidence from observational data, which can make a significant difference in the decision making (Neyarapally et al., 2012).

7. Increased Focus on Structured Benefit-Risk Assessment: The shift towards patient-centric drug development has led to advances in regulatory guidelines for structured benefit-risk (BR) assessment approaches (Hammad et al., 2013; Hammad and Pinto, 2016) to help add transparency to both industry and regulatory decision making process in judging BR profiles. The effort around BR assessment is now expected to be sustained throughout the life cycle of all products. Additionally, conducting quantitative BR assessment using modeling approaches like Multiple-Criterion Decision Analysis (MCDA) (Marsh et al., 2016; Tervonen et al., 2023) is now an option and have been used to incorporate safety findings in the context of products’ benefit by creating a weighting system that would allow the generation of a summary score. This has been used to support regulatory submission for some drugs (Vermersch, et al., 2019) including a study conducted by the FDA for the first time (Lackey et al., 2021). These analytic approaches aim to provide context to safety findings and, ultimately, may facilitate the inclusion of additional information from patient preferences in the assessment and decision-making process (Smith et al., 2016; Janssens et al., 2023). Patient preferences involve gathering information from patients regarding BR tradeoffs, essentially measuring the level of risk patients are willing to accept in exchange for a specific expected benefit. PV physicians need to understand these innovative approaches to avoid assessing safety data in a vacuum, but to do it within the context of products’ benefit and patient needs. This would entail knowing when to request additional BR analyses and how to interpret its results.
8. Evolving Role of Evidence-Based Medicine in Drug Safety: Evidence-based medicine (EBM) has a significant pertinence to drug safety practices.^18^ EBM emphasizes the integration of various sources of evidence, e.g., clinical expertise, patient values, epidemiology, and other available evidence to inform medical decision-making, especially on causality assessment of safety signals (Hammad et al., 2023). Integrating various sources of evidence enhances the objectivity and reliability of safety assessments facilitating more informed decisions making. PV physicians play a crucial role in utilizing EBM principles for critically appraising safety data, conduct meta-analyses, and assess the quality of various sources evidence related to drug safety. Therefore, it requires PV physicians to undergo more focused trainings on the tools of EBM to fully utilize it, including understanding the hierarchy of evidence^19^ as it relates to the strength and value-added of each source of evidence.
9. Significance of Pharmacokinetics and Pharmacodynamics in Drug Safety: PV physicians’ understanding of pharmacokinetics (PK) and pharmacodynamics (PD) is essential for assessing the safety profiles of pharmaceutical products and ensuring safe medication use in clinical practice. Key aspects include drug absorption, distribution, metabolism, receptor binding and response, therapeutic index, and population variability.

For example, determining an effective and safe dose range in a development program depends on appreciating the nuances of the level of receptor occupancy/binding required for acceptable efficacy while minimizing safety concerns. The drug’s therapeutic index^20^, representing the ratio between the minimum effective dose and dose-associated toxicity, helps further define the safe dosage ranges. Understanding drug absorption characteristics enables to further optimize dosing regimens. Food intake, for instance, can significantly increase blood levels of certain drugs (Papasouliotis et al., 2022), impacting the potential for ADRs. Investigating safety signals might also benefit from knowing the drug’s receptor targets, helping determine whether observed ADRs result from on- or off-target effects (Rudmann, 2013), facilitating further investigations to minimize these ADRs.

Other information from PK/PD can also contribute to drug safety efforts. Drug distribution patterns guide achieving therapeutic concentrations at target sites (Rizk et al., 2017), minimizing toxicity. Enzymatic metabolic pathways aid in identifying potential drug interactions and enable adjusting doses based on variations in metabolic capacity.^21^ Drug elimination pathways assist in dose adjustments for patients with impaired renal or hepatic function to prevent drug accumulation and associated adverse effects (Lea-Henry et al., 2018).^22^ Lastly, considering inter- and intra-individual differences in PK/PD, such as due to age, genetic factors, co-medications, and co-morbidities, might enable individualized dosing recommendations to ensure safety across diverse patient populations (Tyson et al., 2020). Nonetheless, the current pharmacological model might not be fully applicable to biologics, gene therapies, and cell therapies and more research is needed in this area.

10. The Use of Meta-Analysis of Randomized Clinical Trials in Drug Safety: Meta-analysis involves the systematic review and statistical synthesis of data from multiple randomized clinical trials (RCTs) to provide a comprehensive assessment of treatment effects. In pharmacovigilance, integrating data from several RCTs becomes necessary sometimes due to the sparse nature of most product related ADRs. This allows for a larger sample size and increases statistical power to detect rare ADRs. By pooling and analyzing data from multiple studies, meta-analyses might provide valuable insights into the overall safety profile of a product, contributing to evidence-based decision-making in drug safety assessments (Sutton et al., 2000; Ioannidis et al., 2004).

However, *post hoc* meta-analyses of RCTs evaluating purported safety findings are increasingly being published, receiving media attention, and influencing clinical and regulatory decision making. Prominent examples include meta-analyses examining risk of cardiovascular events associated with rosiglitazone (Nissen et al., 2010) and tiotropium (Singh et al., 2008), mortality rates associated with cefepime (Kim et al., 2010), and suicidality associated with antidepressant drugs (Hammad et al., 2006; Stone et al., 2009). In this context, it is important to note that *post hoc* meta-analyses evaluating safety issues are subject to biases inherent in retrospective observational studies, and disagreements between meta-analyses and large RCTs have highlighted the need for careful critique of these studies and their limitations in drug safety evaluations (Hammad et al., 2011).

In response to the consequences of poor-quality reporting of RCTs, the Consolidated Standards of Reporting Trials (CONSORT)^23^ Group has added 10 new recommendations about reporting harms-related issues to the standard checklist. This aims to address the need for comprehensive reporting of evidence for ADRs (Ioannidis et al., 2004). Additionally, the CIOMS X guidance^24^ provided comprehensive recommendations on evidence synthesis and meta-analysis in the context of drug safety. Understanding these methodological issues and their implications for data interpretation is crucial for PV physicians. It requires mostly medical judgment in assessing the impact of various methodological aspects, with statistical considerations being just a small part. Building knowledge about the basic concepts of meta-analysis is an important skill for PV physicians to effectively assess the evidence (Hammad et al., 2011).

## 3 Discussion of the evolving role of PV physicians in drug development

PV physicians are expected to leverage their expertise to evaluate safety data and identify potential risks associated with pharmaceutical products. Their responsibilities include actively monitoring and analyzing safety information from various sources to detect adverse events and assess their clinical significance, causality, severity, and frequency. Timely reporting to regulatory agencies is also a crucial aspect of their role. Additionally, they contribute to the development and implementation of risk management strategies, such as appropriate dosing, monitoring, product labeling, risk management plans, and educational materials for patients and healthcare professionals, in order to mitigate identified risks and ensure the safe use of pharmaceutical products.

However, the initial responsibilities and duties of PV physicians traditionally focused primarily on adverse drug reaction reporting and signal detection management in the post-market setting. In early drug development stages, the role of PV physicians was limited, and the burden fell on the clinical development physicians to track and investigate potential safety signals. The skill sets and knowledge needs were limited to that role. PV strategies are now integral to overall drug development plans. So, the role of PV physician has hence evolved to become more holistic, involving safety assessment at every stage of drug development. They are now expected to participate in early preclinical and clinical development, assessing safety profiles, identifying potential safety concerns, and contributing to the design of safety monitoring plans and protocols. By being involved early on, a more thorough evaluation of the investigational product’s safety potential becomes possible, leading to informed decisions regarding the evolving BR balance.

The changing landscape of drug development and safety monitoring, coupled with the expanded role of PV physicians, presents ongoing challenges to their professional development. Comprehensive and specialized training becomes crucial in order to meet these demands. PV physicians are expected to possess a wide array of knowledge and skills, including, for instance, the ability to extrapolate preclinical research findings to predict human experiences. For example, to develop contraception guidelines for a given study, it is critical to have the ability to utilize results from genotoxicity testing, developmental toxicity assessment, on-and off-target mechanisms, drug-drug interactions, characteristics of the studied product, and regulatory guidelines.^25^

Training for PV physicians should expand beyond traditional activities like adverse event reporting and risk management strategies, in order to prepare them for the technical requirements of evolving medicine, drug development, and PV practices. Furthermore, improving critical appraisal skills in evidence-based medicine is important to enhance the ability to evaluate the quality and relevance of safety data from multiple sources. For example, employing a comprehensive approach to causality assessment, incorporating evidence-based medicine tools, allows PV physicians to accurately attribute adverse events to the product versus other factors in a systematic way (Hammad et al., 2023). While on-the-job training is valuable for gaining experience, building a future safety workforce necessitates more targeted efforts, especially considering that medical school curricula may not readily emphasize the development of skills required for successful PV physician roles. Kugener et al. (2021) provided a list of some opportunities for PV education and training. However, academic centers, pharmaceutical companies, and regulatory agencies should increase collaboration to establish hands-on training opportunities through post-doctoral, internship, and fellowship programs, in order to meet the growing demand for well-trained PV physicians.

Lastly, interdisciplinary collaboration is crucial in PV. Collaboration among PV physicians, clinical trial physicians, nonclinical sciences experts, regulatory experts, and data scientists enables a comprehensive and multifaceted approach to identifying, assessing, and managing safety concerns. Each stakeholder brings unique expertise and perspectives to the table, fostering a comprehensive understanding of the product’s safety profile. Clear and concise communication of the reasoning behind the final judgment on safety findings to stakeholders is vital. PV physicians should maintain transparency in decision-making and communicate the inherent uncertainties of the evidence used. They also have a role in informing stakeholders and colleagues about safety-related methodologies and approaches to ensure a shared understanding of drug safety principles and challenges when interpreting pertinent evidence.

## 4 Conclusion

Several key drivers are influencing PV practices in drug development and shaping the required skill sets and knowledge in the field. Overall, these drivers and evolving scope of PV physicians role, emphasize the need for ongoing specialized education, training, and professional development in PV to adapt to the changing landscape of drug development and ensure effective drug safety practices. PV physicians should develop the proficiency in pertinent fields of science to ask the right questions, acknowledge multidisciplinary perspectives, and interpret the overall evidence.

Effective strategies for collecting and evaluating safety data in the initial stages of drug development are vital for enabling prompt, data-driven decisions while fulfilling regulatory mandates without imposing unnecessary strain on the drug development journey. Central to these strategies is the recognition of patients as the primary focus. The decisions taken are fundamentally rooted in safeguarding patients’ wellbeing and ensuring that choices align with the highest standards of patient safety. This is particularly crucial when the balance between benefits and risks for patients is not deemed satisfactory. Moreover, the involvement of a range of multidisciplinary stakeholders is significant in the decision-making process of drug safety. Doing so enhances the robustness of evaluations and aligns decisions more accurately with the intricate realities of drug safety ensuring that patients receive treatments that are both effective and safe.

## Data Availability

The original contributions presented in the study are included in the article/supplementary material, further inquiries can be directed to the corresponding author.

## Footnotes

1
https://www.who.int/teams/regulation-prequalification/regulation-and-safety/pharmacovigilance

2
https://www.ema.europa.eu/en/human-regulatory/post-authorisation/pharmacovigilance/good-pharmacovigilance-practices

3
https://www.fda.gov/drugs/surveillance/postmarket-drug-and-biologic-safety-evaluations

4
https://www.fda.gov/regulatory-information/search-fda-guidance-documents/safety-reporting-requirements-inds-investigational-new-drug-applications-and-babe

5
https://www.ncbi.nlm.nih.gov/books/NBK548212/

6
https://medlineplus.gov/genetics/understanding/precisionmedicine/precisionvspersonalized/

7
https://www.lareb.nl/pub-filepreview?id=21683&p=3564

8
https://link.springer.com/article/10.1007/s40264-018-0719-2#Sec287

9
https://www.fda.gov/news-events/fda-voices/fda-releases-two-discussion-papers-spur-conversation-about-artificial-intelligence-and-machine

10
https://clinicalinfo.hiv.gov/en/glossary/cmax#:∼:text=Peak%20Concentration,Cmin

11
https://www.ema.europa.eu/en/human-regulatory/marketing-authorisation/pharmacovigilance/risk-management/risk-management-plans

12
https://www.fda.gov/drugs/drug-safety-and-availability/risk-evaluation-and-mitigation-strategies-rems

13
https://www.fda.gov/regulatory-information/search-fda-guidance-documents/safety-reporting-requirements-inds-investigational-new-drug-applications-and-babe

14
https://cioms.ch/working-groups/working-group-xii/

15
https://www.ema.europa.eu/en/documents/scientific-guideline/guideline-good-pharmacovigilance-practices-gvp-module-viii-post-authorisation-safety-studies-rev-3_en.pdf

16
https://www.fda.gov/drugs/postmarket-requirements-and-commitments/postmarketing-requirements-and-commitments-legislative-background

17
https://cioms.ch/working-groups/real-world-data-and-real-world-evidence-in-regulatory-decision-making/

18
https://www.ncbi.nlm.nih.gov/books/NBK52819/

19
https://www.cebm.ox.ac.uk/resources/levels-of-evidence/ocebm-levels-of-evidence

20
https://clinicalinfo.hiv.gov/en/glossary/therapeutic-index-ti#:∼:text=A%20ratio%20that%20compares%20the,the%20safer%20the%20drug%20is.

21
https://www.fda.gov/drugs/drug-interactions-labeling/healthcare-professionals-fdas-examples-drugs-interact-cyp-enzymes-and-transporter-systems

22
https://www.fda.gov/media/78573/download

23
https://www.equator-network.org/reporting-guidelines/consort/

24
https://cioms.ch/publications/product/evidence-synthesis-and-meta-analysis-report-of-cioms-working-group-x/

25
https://www.ema.europa.eu/en/documents/other/safety-working-party-recommendations-duration-contraception-following-end-treatment-genotoxic-drug_en.pdf
